# The Interaction of Polymorphisms of IL10 and DBH Was Associated with General Symptoms of PANSS with TD in Chinese Han Schizophrenic Patients

**DOI:** 10.1371/journal.pone.0070963

**Published:** 2013-08-07

**Authors:** Hongqiang Sun, Fan Wang, Hongzhen Fan, Quanzhi Yan, Kaiyan Cui, Wei Yuan, Fushuai Zhao, Lili Zhao, Jie Yuan, Fude Yang, Thomas R. Kosten, Xiang Yang Zhang

**Affiliations:** 1 Beijing Hui-Long-Guan Hospital, Peking University, Beijing, China; 2 School of Basic Medical Sciences, Inner Mongolia Medical University, Hohhot, Inner Mongolia, China; 3 Department of Psychiatry, Shandong Mental Health Center, Jinan, Shandong Province, China; 4 Department of Psychiatry, The Sixth Hospital of Changchun City, Changchun, Jilin Province, China; 5 Department of Psychiatry, The Hulunbuir City Mental Health Center, Hulunbuir, Inner Mongolia, China; 6 Menninger Department of Psychiatry and Behavioral Sciences, Baylor College of Medicine, Houston, Texas, United States of America; Peking University, China

## Abstract

**Objective:**

Tardive dyskinesia (TD) is a human hyperkinetic movement disorder as a result of potentially irreversible long-term chronic first-generation antipsychotic medications. Unfortunately, mechanisms involved in the development of TD have been poorly understood. Previous studies have indicated that some genetic polymorphisms of immune system and dopamine beta-hydroxylase (DBH) genes may be involved in the pathogenesis of TD. Rs1800872 and rs72393728 are located on the promoter of interleukin-10 (IL10) and DBH gene, respectively. The genetic association between the rs1800872 and TD is unclear. Previous studies have indicated that genetic variations of IL 10 and DBH are implicated in the positive and negative symptoms in schizophrenia. However, the interaction of two variations with severity of TD and symptoms of schizophrenic patients with TD has not been reported. The present study investigated whether these variations and their interaction were associated with clinical phenotypes of TD with schizophrenia in a genetically homogeneous northern Chinese Han population.

**Methods:**

Rs1800872 and rs72393728 were genotyped in schizophrenic patients with TD (*n* = 372) and without TD (NTD; *n* = 412). The Abnormal Involuntary Movement Scale (AIMS) and Positive and Negative Syndrome Scale (PANSS) were applied to assess the severity of TD and psychopathology of schizophrenia, respectively.

**Results:**

The allele and genotype frequencies of rs1800872 and rs72393728 did not significantly differ between TD and NTD patients (*p>*0.05). No significant difference was found in the AIMS total score among the genotypes of two loci (*p>*0.05). Interestingly, the interaction of rs1800872 and rs72393728 showed a significant association with the PANSS general score (*p* = 0.011), and a trend toward to the PANSS total score (*p* = 0.055).

**Conclusion:**

These findings suggest that the interaction of rs1800872 and rs72393728 variants may play a role in psychopathology of the general symptoms on PANSS in schizophrenic patients with TD in a northern Chinese Han population.

## Introduction

Tardive dyskinesia (TD) is an iatrogenic human hyperkinetic movement disorder and potentially irreversible long-term adverse effect of treatment associated with chronic first-generation antipsychotic medications [1] and is characterized by involuntary, repetitive, purposeless movements. Patients with schizophrenia are vulnerable to the development of TD after antipsychotic treatment [2].

Cytokines are key proteins involved in the immune system activation. Several studies have demonstrated alterations of the level of interleukins in schizophrenia [3]. An immune response shifting from Type 1 (including one cytokine interleukin-2(IL2)) to Type 2 (including one cytokine interleukin-10(IL10)) happens in schizophrenia [4]. IL10, as an important immunoregulatory cytokine, is located on a region reported to be related to schizophrenia in genetic association studies [5]. TD patients have different serum IL2 levels from non-TD patients, and the concentration of IL2 is associated with the negative symptoms of schizophrenia [6]. Level of IL10 increasing in the cerebrospinal fluid of schizophrenics has been described before [7], [8]; a previous study has mentioned that a strong relationship between an increased IL10 level in cerebrospinal fluid and negative symptoms of schizophrenia was observed [9]. Some polymorphisms on IL10 gene are associated with susceptibility to the development of schizophrenia [10]–[12]. The C to A nucleotide exchange of rs1800872 gives rise to increased IL10 gene promoter activity, suggesting its role as a repressor element of the C allele [13].

Dopaminergic system abnormality in nature in schizophrenia is presynaptic involving in dopamine synthesis and baseline synaptic dopamine density [14].Therefore, dysfunction in the dopaminergic system plays a key role in the pathophysiology of schizophrenia [15]. A deficit in DA transmission at dopamine D1 receptors in the prefrontal cortex might contribute to the cognitive impairments and negative symptoms of schizophrenia [15], and genetic variation of D2 receptor are associated with clinic response to antipsychotic drugs[16]–[18]. Dopamine beta-hydroxylase (DBH), which is responsible for catalyzing the conversion of dopamine to norepinephrine, is specifically expressed in adrenergic and noradrenergic neurons in the central nervous system [19]. Previous reports have shown that DBH involved in psychotic symptoms of several mental disorders, such as schizophrenia [20], attention deficit hyperactivity disorder [21], and post-traumatic stress disorder [22], and most of all, levels and activity of the DBH in the plasma and cerebrospinal fluid are closely related biochemical phenotypes in schizophrenia [23]. The rs72393728 is locating on 4.5 kilobases upstream of the transcriptional start site for the DBH gene, and the deletion(*de*l) allele is associated with the regulation of promoter activity leading to lower DBH enzyme levels and activity in plasma and cerebrospinal fluid [24], [25]. Moreover, rs72393728 is reported to be associated with cognitive function and depression [26], [27].Taken together; the previous studies have suggested that two polymorphisms played an important part in psychopathological symptoms.

The susceptibility of schizophrenia with degree of genetic relationship is too complex to be explained by a single gene or the sum of effects of several such genes, and schizophrenia might be an end result of a complex interaction between thousands of genes and multiple environmental factors. The overall genetic contribution to schizophrenia may be large, but none of them on their own could cause schizophrenia. Thus, the inheritance pattern of schizophrenia suggests that each of multiple genes with small effect interacts with one another and with environmental factors nonlinearly to influence susceptibility [28]. Patients with TD receive long-term medication of antipsychotics; the genetic pattern of TD might be the same as that of schizophrenia. It would have been too difficult to detect these variations through single polymorphism analysis, and complex disorders are assumed to be explained by the interaction of multiple genetic polymorphisms and environmental risk factors with complicated mechanisms, therefore, demonstration of the usefulness of the approach to detect and identify gene×gene and/or gene×environment to schizophrenia has been one of the most important and challenging topics to us.

In this current study, we discussed whether the two vitiations, rs1800872 and rs72393728 on IL10 and DBH genes and their interaction contributed to the susceptibility and the development of TD and the schizophrenic patients’ psychopathology measured by the Positive and Negative Syndrome Scale (PANSS) in a group of northern Chinese Han schizophrenic patients.

## Materials and Methods

### Ethics Statement

This project was approved by the Institutional Review Board of Beijing Hui-Long-Guan Hospital. Informed written consent was obtained from all participants before taking part in this study after given a verbal and written complete and detail description of the study. The clinical staff explained the nature of the subjects, risks, benefits of this study. Meanwhile, the participants were informed to opt not to join the study without any influencing on the treatment in the hospital. If a subject could not understand these issues due to the status of mental health impaired, he/she was not asked to take part in this study. If the participant had a possible compromised capacity to consent, his/her next of kin, care takers or guardians consented on the behalf of the participant signed the additional written informed consent.

### Subjects

Seven hundred eighty-four schizophrenic inpatients (*n* = 664 males, *n* = 120 females) took part in this study. Participants were all northern Chinese Han population from Beijing Hui-Long-Guan Psychiatric Hospital and Rong-Jun Hospital in Hebei province. Diagnoses met the criteria for chronic schizophrenia of *Diagnostic and Statistical Manual of Mental Disorders*, 4th edition. All of them received ongoing stable dose of oral antipsychotic drugs at least one year, when they were recruited. Antipsychotic drug treatment was composed mainly of monotherapy with clozapine (n = 364), risperidone (n = 164), perphenazine (n = 35), sulpiride (n = 42), chlorpromazine (n = 58), haloperidol (n = 27), aripiprizol (n = 24), quetiapine (n = 28) and other typicals and atypicals (n = 42). Mean antipsychotic dose being in chlorpromazine equivalents was 462±452 mg/day. The patients had been on their respective medication for 23.29±10.46 years at the time of the investigation. All patients were chronic type with mean duration of illness for 24.58±9.379 years and between 19 and 73 years old. Subjects without a history of drug abuse or dependence, with the exception of alcohol and nicotine using, took part in this study.

### Clinical Measures

A questionnaire was used to collect general information, demographic characteristics, psychological condition, and medical history or each patient. Other information was obtained from medical records.

Four experienced trained psychiatrists who did not know about the clinical status of the patients assessed the severity of TD using the Abnormal Involuntary Movement Scale (AIMS) [29]. The details of the diagnosis were described as the previous study [30]. A total of 372 TD patients were included in the study (n = 344 male, n = 28 female). The rest of total of 412 without TD (NTD) patients were included (n = 320 male, n = 92 female).

The same four psychiatrists assessed the psychopathology of all participants using the PANSS at begin with all the subjects entering into this present study. The four psychiatrists simultaneously received training ahead of the study. All of the assessments were completed prior to the laboratory experiments.

### DNA Extraction and Genetic Analysis

Five milliliter peripheral blood was used to extract genomic DNA with a salting-out method [31]. The two variations were genotyped using polymerase chain reaction restriction (PCR) and the PCR product of rs1800872 (−592C>A) need to be dealt with fragment length polymorphism (RFLP) as described below. All samples were genotyped duplicatedly.

The primers and PCR condition of rs1800872 and rs72393728 were applied as the reference [32], [33]. The 412 bp PCR product of rs1800872 was digested with 5U RsaI restriction enzyme (New England Biolabs, Beverly,MA) and then underwent electrophoresis in 2% agarose gel stained with ethidium bromide, and this products remained intact (allele C) with 412 bp,and two fragments of 236 bp and 176 bp fragments for allele A [32]. The Products for the insertion/deletion (*ins/del*) genotypes were *del* homozygote (144 bp), and *ins* homozygote (163 bp) with 3% agarose gel stained with ethidium bromide [33].

### Statistics Analysis

The SHEsis project [34] was used to calculate the allele and genotype frequencies, Hardy-Weinberg Equilibrium and differences in allele and genotype frequencies between groups. Chi-square test or analyses of variance (ANOVA) were used to calculate interactions between (i) genotype and gender and (ii) genotype and age, and two-way two-sample *t*-test for continuous variables was analyzed for testing group differences. Independent variables, including genotype, sex, age, hospitalization time, and age of onset, were analyzed using regression analysis for TD and NTD patients as the dependent variables. Logistic regression was performed to adjust underlying interference factors for TD using TD as a dependent variable and genotype, sex, age, age at onset, antipsychotic type (atypical vs. typical drug) and duration of antipsychotic treatments as independent and dose of drugs (equivalent to chlorpromazine). GMDR project was used to calculate the interactions of polymorphisms and continuous variable, which is a platform of nonparametric and genetic model-free alternative to linear or logistic regression to detect and characterize nonlinear interactions among discrete genetic and environmental attributes [35]. Missing data were omitted separately from analysis by software. These analyses were carried out in all samples and then in the TD cases. The reported *p*-values were at the significance level of 0.05. We calculated *p*-values for interaction models based on 1,000 permutations.

## Results

Patients did not show a departure from Hardy-Weinberg Equilibrium in TD group for two polymorphisms (*p* = 0.752 and *p* = 0.751 for rs1800872, respectively in NTD and in TD group; *p* = 0.603 and *p* = 0.930 for rs72393728, respectively in NTD and in TD group). The AA, AC and CC genotypes of rs1800872 were found in 284 (39.78%), 337 (47.20%) and 93 (13.03%) of the 714 patients, respectively, and the genotype data of 70 samples were missing. The *ins* homozygote, *ins/del* and *del* homozygote of rs72393728 were found in 253 (33.64%), 370 (49.20%) and 129 (17.15%) of the 752 patients, respectively, and the genotypes of other 32 samples were not genotyped. There was no significant association found the between rs1800872, rs72393728 and TD (*p* = 0.999 and *p* = 0.895, respectively).

Demographic variables and PANSS and AIMS scores in each genotype of rs1800872 and rs72393728 are shown in Table 1 and Table 2, respectively. The PANSS subscores and total scores and AIMS scores for the three genotypes were not significantly different (both *p*>0.05 for each locus). Continuous variables were analyzed using a one-way ANOVA for each genotype with respect to sex, age and age at onset for both variations (*p*>0.05). In addition, No interaction was found between gender and genotype (*χ^2^* = 2.374, df = 2, *p* = 0.305 and *χ^2^* = 0.948, df = 2, *p* = 0.623, respectively.) or between age and genotype in both two loci (F = 1.909, df = 2, *p* = 0.149 and F = 1.933, df = 2, *p* = 0.145, respectively.). Total AIMS scores were 2.66±3.08 for AA, 2.65±3.20 for AC and 2.38±2.48 for CC (F = 0.314, *p* = 0.731) in rs1800872. Total AIMS scores were 2.67±2.86 for *ins/ins*, 2.71±3.39 for *ins/del* and 2.33±2.95 for *del/del* (F = 0.711, *p* = 0.491) in rs72393728.

**Table 1:** Demographic characteristics of the patients with schizophrenia (*n* = 714) with the rs1800872 polymorphism.

		Genotype		
		A/A	A/C	C/C
Sex (male/female)		241/43	290/47	74/19
Age (years)		48.46±9.77	47.75±9.29	46.21±10.23
Age at onset (years)		23.90±5.95	23.12±5.09	23.09±4.80
**PANSS**				
Total score		59.79±14.25	60.49±15.17	58.65±13.01
Positive score		11.62±4.79	11.84±5.15	11.98±5.52
Negative score		23.02±7.95	23.06±8.12	21.49±7.68
General score		25.55±7.73	25.56±6.02	24.90±5.77
AIMS score		2.66±3.08	2.65±3.20	2.38±2.48

**Table 2:** Demographic characteristics of patients with schizophrenia (*n* = 752) with the rs72393728 polymorphism.

		Genotype		
		*ins/ins*	*ins/del*	*del/del*
Sex (male/female)		214/39	308/62	112/17
Age (years)		48.60±10.04	48.01±9.55	46.57±8.64
Age at onset (years)		23.43±5.83	23.44±5.37	23.33±4.25
**PANSS**				
Total score		60.49±14.99	59.86±15.16	60.35±13.61
Positive score		11.80±5.05	11.60±5.03	12.13±4.94
Negative score		22.88±8.18	22.96±8.42	23.33±7.56
General score		25.82±6.33	25.23±5.97	25.63±9.10
AIMS score		2.67±2.86	2.71±3.39	2.33±2.95

The differences in demographic and clinical variables between TD (*n* = 372) and NTD group (n = 412) are shown in Table 3. Significant differences in age (*t* = 3.480, *p* = 0.001) and hospitalization time (*t* = 4.489, *p*<0.0001) were found between TD and NTD groups. There were more male TD patients (51.8%, 344 of 664) than female TD patients in the whole population (23.3%, 28 of 120; *χ^2^* = 33.05, *p*<0.0001). However, no difference was found in age at onset between the two groups (*t* = −0.391, *p* = 0.696). The negative symptom scores on the PANSS were found that there was significant different between TD and NTD subjects (*p*<0.0001).

**Table 3:** Characteristics of TD and NTD patients.

Characteristic	TD (*n* = 372)	NTD (*n* = 412)	*t* or *χ^2^*	*p*
Sex (male/female)	344/28	320/92	33.05	0.000
Age (years)	49.30±8.92	46.92±10.02	3.480	0.001
Age at onset (years)	23.36±4.97	23.51±5.73	−0.391	0.696
Hospitalization time(years)	24.62±8.86	21.55±10.10	4.489	0.000
**PANSS**				
Total score	61.22±14.04	59.42±15.27	1.701	0.089
Positive score	11.54±4.79	12.01±5.27	−1.305	0.192
Negative score	24.54±7.72	21.64±8.24	5.021	0.000
General score	25.34±7.13	25.78±6.24	−0.915	0.360

Logistic regression analyses were calculated for some demographic variables, the clinical variables, and the genotypes of rs1800872 and rs72393728 to determine whether these variables were risk factors for TD. To judge the relative importance of the selected independent variables, rs1800872 and rs72393728 genotype was represented as 2 dummy variables, each coded with the number (0, 1, or 2) of alleles, respectively, in each genotype. Sex (male; *p* = 0.000, OR = 0.266, CI = 0.156–0.451; male; *p* = 0.000, OR = 0.286, CI = 0.173–0.473, respectively.), and PANSS negative symptom scores (*p* = 0.015, OR = 1.028, CI = 1.005–1.050; *p* = 0.018, OR = 1.025, CI = 1.004–1.047, respectively.) were risk factors of TD on rs1800872 and rs72393728 respectively. No association was found between genotype and TD (respectively, *p* = 0.632 and *p* = 0.471 to rs1800872 and *p* = 0.994 and *p* = 0.782 to rs72393728, respectively.).

**Figure 1:**
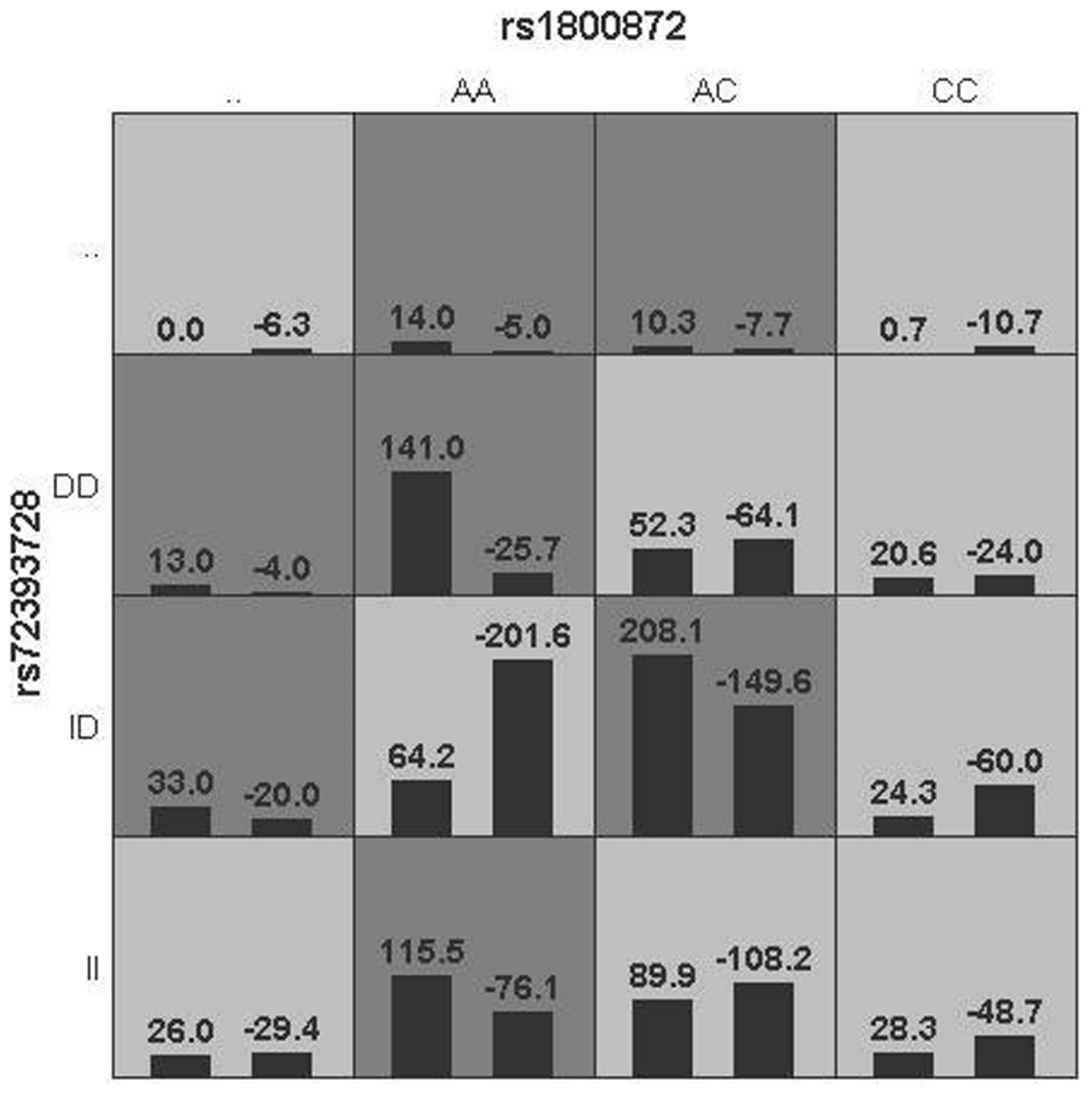
The rs1800872×rs72393728 interaction showed a significant association with general scores on the PANSS (p = 0.011) in the TD group.

To explore the interaction of two loci to the severity of TD and the psychopathology of schizophrenia, we analyzed the influence of rs1800872×rs72393728 to the score of AIMS and PANSS total scores and subscores with GMDR algorithms to detect the top potential interaction between the analyzed variations and distribution of high-risk and low-risk genotypes in the best two-locus model, dark gray and light gray boxes stood for the high- and low-risk factor combinations, respectively. Left bars within each box represented TD while the right bars represented NTD. We found that the interplay had effect on with general symptom scores on the PANSS, and the OR for the high-risk genotype combination (AC)-(*ins/de*l) was 2.758 (95%CI: 1.372–5.542, *p* = 0.011, see Figure1) in this two-locus (rs1800872–rs72393728) model.

**Figure 2:**
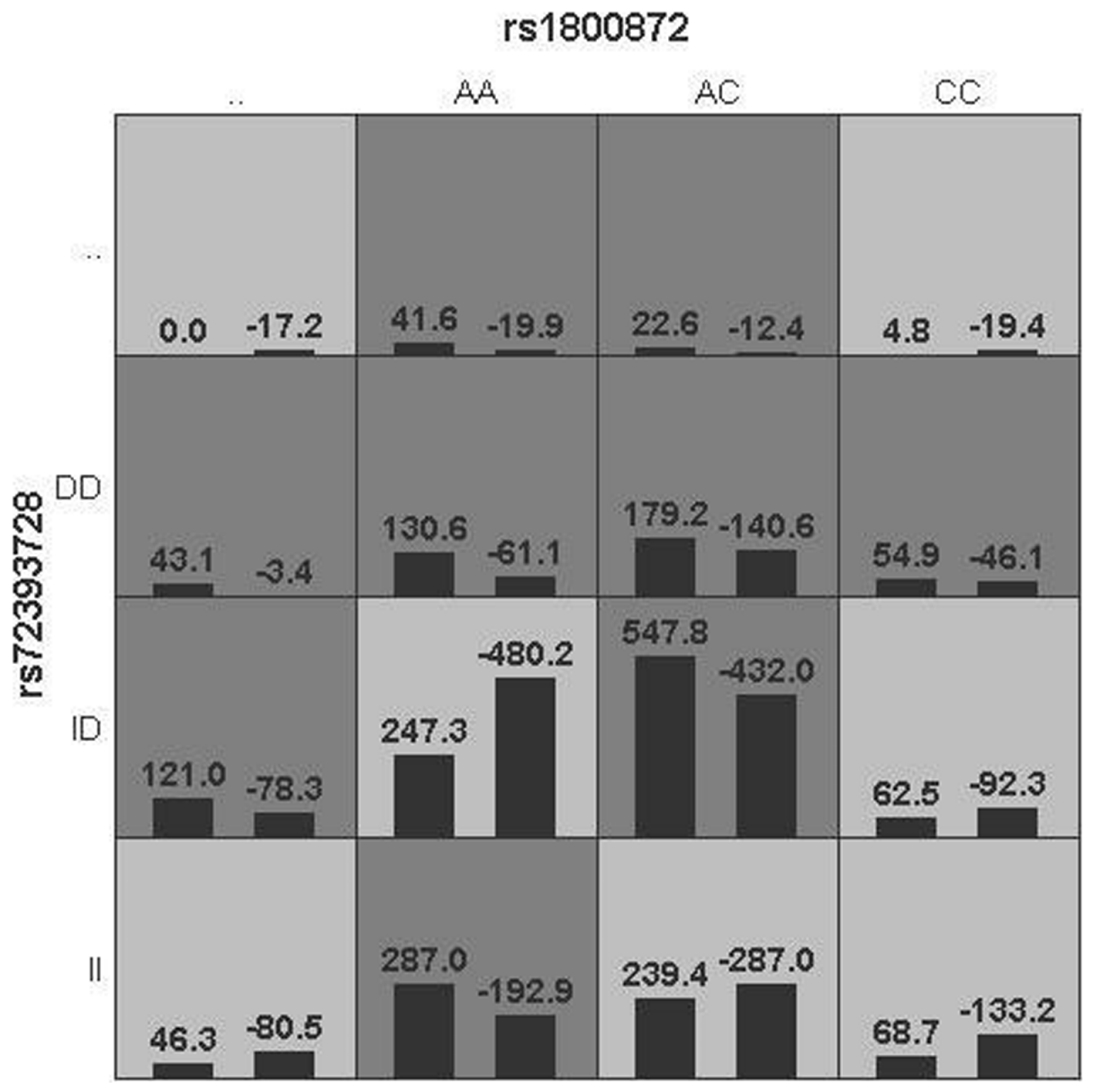
The rs1800872×rs72393728 interaction showed a trend toward significance for total scores on the PANSS in the TD group (p = 0.055).

The same interaction showed a trend toward the significant association with total scores on the PANSS (*p* = 0.055, see Figure 2) in TD group. The former interaction model of the genotypes of AC and *ins/de*l suggested that it was a risk factor for general symptom symptoms on the PANSS of TD patients, but only a potential risk factor for total scores on the PANSS of the same group.

## Discussion

It is well known that the best methodology to detect interaction remains controversial. Using the GMDR method followed by conventional statistical analysis, the best high-risk gene×gene model identified was a two-locus genotype combination in this study.

To our knowledge, this study is the first report that the polymorphisms interaction showed a significant association with the PANSS general symptoms in TD patients. In this current study, we genotyped rs1800872 and rs72393728 of DBH gene for investigating whether the variations and their interaction were susceptibility to TD, the development of TD and the schizophrenic patients’ psychopathology in genetic association study. The results were followed: (1) No significant association was found between TD and rs1800872 and rs72393728 on DBH gene. (2) Age (older age), sex (male), hospitalization time (longer) and PANSS negative symptoms were major risk factors for TD. (3) The model of rs1800872×rs72393728 may be a risk factor for general symptom scores on the PANSS and for a trend toward significance with total scores on the PANSS of TD patients.

We did not detect significant association between rs1800872, rs72393728 and schizophrenia in our research. Older age, male, longer hospitalization time and PANSS negative symptoms were major risk factors for TD, which is consistent with our previous investigation [30]. Rs1800872 and rs72393728 were reported to be significantly different between schizophrenic patients and controls [12], and associated with cognitive function and depression [26], [27], respectively. IL10 is a Th2 anti-inflammatory cytokine that participates in the regulation of the immune response at several levels [36], which has immunosuppressive activities including the ability to downregulate the expression of macrophage costimulatory molecule. Changed DBH activity leads to misregulation of inflammation in the brain. The mechanisms that control inflammation in the brain are different from those in the periphery. The noradrenergic network plays an important part in the former control system. Increased levels of noradrenaline reduce expression of pro-inflammatory cytokines [37]. There may be a biological connection between IL10 and DBH in functions.

For further explanation for biological significance of interaction, we founded that information of location for the variations, which is noteworthy that rs1800872 and rs72393728 (both located on the promoter) might affect on the expression level of proteins [38]. The strong interaction observed suggested a possible interaction as both genes may be involved in TD. It has been reported that the A allele of rs1800872 was associated with decreased IL10 expression in the in vitro experiment [39], and the CC genotype was associated with higher IL10 production than other genotypes [40]. The *de*l allele is associated with lowering DBH levels and enzyme activity in plasma and cerebrospinal fluid [24], [25]. With the exception of the above interaction (rs1800872×rs72393728), the present data showed that there was additive interaction tendency between rs1800872 and rs72393728 on the PANSS total scores with TD might be due to this interaction with TD on notably the PANSS general symptoms. What the genotypes AC and *ins/de*l might be easy to regulate the patterns of genes expression could result in this specific interaction showing the significance, because of the heterozygote in two genes. The two variations that we genotyped gave us an interaction model of heterozygous, and IL10 and DBH have been identified to influence negative and positive symptoms of schizophrenia, respectively [9], [41]. Therefore, we did not found any interaction of influence negative and positive symptoms due to the combination of two heterozygous genotypes. However, the real biological significance of rs1800872×rs72393728 remains unclear.

There are several limitations needed to be mentioned in this current study. First, it is important to think about the sample size to detect the effect when initiating a genetic study, but our subjects were relatively large, and needs more participants generally. And the demographic factors were not well-matched, such as gender, age and hospitalization time between TD and NTD groups. Second, although the Chinese around Beijing and Hebei province might be ethnically relatively homogenous, minute differences still exist [42]. Stratification of hidden population in our samples could still be confounders. Third, just two variations were genotyped in this study, which did not fully cover the two genes totally. Rs1800872 and rs72393728 are both located on upstream of gene, respectively, where epigenetic mechanisms may be partly involved in regulation of gene expression. More polymorphisms of these two genes, preferably tags for the Chinese population, should be genotyped to provide more information. Fourth, second-generation antipsychotics have been showed a reduced risk for TD in schizophrenia, compared with the first-generation antipsychotic according to long-term studies presented involving enough patients [43]. In this study, the participants received different medications of first-generation or second-generation antipsychotics, which might affect the susceptibility to TD. If all subjects received unified one kind treatment, there will be more accurate data to analyze. Fifth, the important factor affecting the statistical power and this sample size of the study is the strength of the association between the exposure and the outcome; the typical measure of the strength of an association is the risk ratio. A previous study has given an ambiguous epidemiological risk ratio range for TD among schizophrenic patients [44], therefore we did not calculate an accurate statistic power. However, based on the minor allele frequency, the power is not enough high, and we need more subjects to verify these results. Finally, the given interaction model by GMDR calculating are hard to be understood. A genetic interaction between some genes implies a functional relationship between those genes. Genetic interactions should reveal specific dependencies within the genetic network and can provide a powerful method for functional characterization. However, either negative or positive functional explanation of a single genetic interaction is not obtained.

However, it is important to derive a reasonable biological interpretation of identified gene×gene. To strengthen the reliability of the interaction founded in our study, we conducted analysis by existing biological resources of bioinformatics to aid the biological interpretation of the identified gene×gene. In other words, the goal of this step is to determine known biological evidence that supports the identified gene×gene. A previous study suggested that if two genes are involved in generating the variability of a phenotype together, biological interaction between them or their products must be involved [45]. Through the network graph analysis, the protein-protein/gene-gene interacts and transcription factors targets prediction analysis did find possible associations between DBH and interleukin-34 that could play an important role central nervous system [46] by the online project COXPRESdb [47], and the previous report has implied that interleukin-34-macrophages had relations with IL10 exhibit [48]. However, another point of view is that the existence of a genetic interaction between two genes does not mean the two gene products interacting physically each other, or the genes temporally coexpressing; it simply suggests that some kind of functional relationship is shared [49].

On one hand, there is a mass of interactions mechanisms and phenomena in organism, with feedback regulation at several levels, such as the nucleotide molecular, regulatory factor, signal transduction and metabolic and physiological processes. It seems that the biological system is a network of interplays. On the other hand, interactions might be a consequence of biological evolutionary processes [50]. Phenotypes of genetic unstable expression caused by a mass of interaction factors and active homeostasis might be taken for a response to natural selection and other selective forces in evolution. And the result of effects of factor levels might generate a marginal correlation between the levels of each factor separately and the phenotypes.

Although the result in the current study may be of limited value for clarifying the underlying pathogeny, more interaction models that identify the potential loci of disorders should generate more power for detection of genetic effects. However, models of gene×gene/gene×environment given by statistical methods are likely to accelerate the discovery and identifying of genetic variants of biological system network, which might be feasible to help understand the real biological explanations for various physiological effects of the researched polymorphisms further. However, whether these polymorphisms are functional and all factors interplay need be confirmed further by experimental evidence finally.
